# CMV retinitis in China and SE Asia: the way forward

**DOI:** 10.1186/1471-2334-11-327

**Published:** 2011-11-24

**Authors:** David Heiden, Peter Saranchuk

**Affiliations:** 1 Department of Ophthalmology, California Pacific Medical Center, San Francisco, CA, USA; 2 Seva Foundation, Berkeley, CA, USA; 3 South African Medical Unit, Operational Centre Brussels, Medicines Sans Frontieres, Cape Town, South Africa

## Abstract

AIDS-related CMV retinitis is a common clinical problem in patients with advanced HIV/AIDS in China and Southeast Asia. The disease is causing blindness, and current clinical management, commonly characterized by delayed diagnosis and inadequate treatment, results in poor clinical outcomes: 21% - 36% of eyes with CMV retinitis are already blind at the time the diagnosis is first established by an ophthalmologist. CMV retinitis also identifies a group of patients at extraordinary risk of mortality, and the direct or indirect contribution of extra-ocular CMV disease to AIDS-related morbidity and mortality is currently unmeasured and clinically often overlooked. The obvious way to improve clinical management of CMV retinitis is to screen all patients with CD4 counts < 100 cells/μL with indirect ophthalmoscopy at the time they first present for care, and to provide systemic treatment with oral valganciclovir when active CMV retinitis is detected. Treatment of opportunistic infections is an integral part of HIV management, and, with appropriate training and support, CMV retinitis screening and treatment can be managed by the HIV clinicians, like all other opportunistic infections. Access to ophthalmologist has been problematic for HIV patients in China, and although non-ophthalmologists can perform screening, sophisticated ophthalmological skills are required for the management of retinal detachment and immune recovery uveitis, the major complications of CMV retinitis. CMV retinitis has been clinically ignored, in part, because of the perceived complexity and expense of treatment, and this obstacle can be removed by making valganciclovir affordable and widely available. Valganciclovir is an essential drug for developing successful programs for management of CMV retinitis in China and throughout SE Asia.

## AIDS-related CMV retinitis is a common problem in China

Cytomegalovirus (CMV), a member of the herpesvirus family, was a familiar, potentially blinding and lethal opportunistic infection in high income countries prior to the introduction of highly active anti-retroviral therapy (HAART). As the burden of the epidemic shifted to middle and low income countries, CMV retinitis, the most common AIDS-related clinical manifestation of CMV disease, became the "neglected disease of the AIDS pandemic" [1]. The current report by Shi, et al [2] brings important attention to this problem in China: CMV retinitis was found in 16.8% (19/113) of HIV-infected inpatients having CD4 counts < 50 cells/μL (the high-risk group). The data from Shanghai complement accounts of CMV retinitis from Beijing [3] and Guangzhou [4], and is consistent with our personal observation of a similar burden of CMV retinitis during training projects and clinical work in HIV clinics and infectious disease hospitals between 2006-2010, in Hubei province and the Autonomous Regions of Guangxi and Xinjiang. Although the authors appropriately suggest that we acquire better epidemiologic data, the key point is that there is adequate evidence that AIDS-related CMV retinitis is a common clinical problem in China, as in SE Asia [1].

## Blindness

The visual consequences of CMV retinitis are disastrous. In the current report [2], 20.8% (5/24) of eyes with CMV retinitis were blind at diagnosis. In reports from Thailand [5,6], 31.2% (92/295) and 31.5% (165/523) of eyes were blind at diagnosis, and in a report from multiple sites [1], 35.9% (37/103) of eyes with CMV retinitis were blind.

This staggering level of blindness is actually worse than the simple numbers suggest, for three reasons. First, the vision loss resulting from CMV retinitis is commonly severe, with one prior report showing only hand motion (HM) vision or worse in 33/37 (89.2%) of eyes blinded by CMV [1]. The World Health Organization (WHO) defines blindness as < 3/60 visual acuity, which at the bedside equates to an inability to count fingers at a distance of 10 feet. In reality, blindness includes several distinct and functionally different groups, and the majority of patients that meet the WHO definition still retain enough vision to manage the simple activities of daily living (dressing, feeding, navigating to the bathroom, etc.). But with HM vision or worse, a person requires "sight substitution," which in resource-limited settings means that another person must almost continually provide assistance; patients with this level of visual disability struggle to survive.

Second, despite the therapeutic optimism of the current report [2], CMV infection causes irreversible full-thickness retinal necrosis, and most vision loss from CMV is permanent (although some improvement may occur with resolution of adjacent retinal edema and clearing of inflammatory debris from the vitreous cavity). In contrast, vision loss from cataract, the most common cause of blindness, may be cured and vision fully restored.

Third, again unlike cataract, AIDS-related CMV blindness occurs at a young age. Traditionally, epidemiologic studies for blindness do not even survey the population less than 40 years of age because blindness before that age has been so rare. But now, in young patients receiving effective treatment for HIV/AIDS, there is a progressive accumulation of unfortunate people who are permanently and profoundly blind. In an area hard-hit by HIV in northern Thailand, CMV retinitis was found to be the second most common cause of blindness (following cataract) in a consecutive series of all patients referred to a University eye clinic, even though it occurred only in patients infected with HIV, which represented less than 2% of the general population [7].

## Mortality

The mortality described in this report from Shanghai is even more striking than the incidence of blindness: 30.4% (7/23) of patients with CMV retinitis died within 2 weeks. We observed a similar mortality rate in Yangon, Myanmar (unpublished abstract, presented at the annual meeting, Association for Research in Vision and Ophthalmology (ARVO), May 4, 2010: **Outcomes for a 42 patient CMV Retinitis cohort from the slums of Rangoon**. *D. Heiden, N.J.S. London, N. Tun, F. Smithuis.)*: in a sub-group of patients diagnosed with active CMV retinitis and followed 30 months: 31.8% (7/22) died, 27.3% (6/22) were confirmed alive, and 40.9% (9/22) were lost to follow-up, including 3 within the first month who almost certainly died as well.

The relationship between CMV infection and mortality, a subject of intense interest in the "pre-HAART era" in the United States and Western Europe, now receives little attention. Autopsy data is rarely acquired, and it is often forgotten that disseminated CMV infection was found in 38-59% of patients dying of AIDS [8,9]. In patients who die of AIDS, the precise reason for a fatal outcome may be difficult to determine, although the patient with transverse myelitis reported by Shi almost certainly died of CMV [10]. The evidence linking CMV infection and excess mortality is complex. Western data has shown that CMV is a co-factor in AIDS progression [11], and that CMV viremia [12,13] and retinitis [14] are powerful predictors of mortality. A report from Cambodia demonstrated that CMV viremia was associated with a doubling of mortality [15].

## Early diagnosis

Dismal outcomes are inevitable if CMV retinitis is diagnosed late. We strongly support Shi's assertion that retinal screening should be part of a ***standard initial evaluation ***for all individuals presenting with CD4 counts below 100 cells/μL, and we strongly agree about the importance of screening asymptomatic patients. This is consistent with previous evidence and recommendations [16].

CMV retinitis is a clinical diagnosis. The "gold standard" is examination of the entire retina through a fully dilated pupil with an indirect ophthalmoscope by an experienced observer. The characteristic features of CMV retinitis are easily appreciated. Thus diagnosis can be established rapidly and at minimal cost in any consultation room or hospital ward, without need for special testing, eye clinic, or ophthalmologist. Management of CMV retinitis can in fact be integrated into the primary care of the patient with AIDS, just like all other opportunistic infections, and a recent report from Myanmar describes successful diagnosis and treatment of CMV retinitis by non-ophthalmologist HIV clinicians at the primary care level [17]. Innovation is also taking place in Thailand, where ophthalmologists are exploring the use of telemedicine to screen for CMV retinitis at the primary care level [18].

Early diagnosis is the most effective way to reduce the blinding complications of CMV retinitis. The risk of both retinal detachment and immune recovery uveitis (IRU) increase as infection advances, and is proportional to the size of the retinal surface area involved [19,20]. Both of these complications can be complex and expensive to treat, and often may have poor outcomes, even with the best treatments that modern medicine can offer.

Early diagnosis of CMV retinitis depends on patients having access to care; unfortunately the stigma attached to HIV in China has commonly excluded patients from ophthalmological care, particularly surgery [21]. (We have seen a patient left permanently blind because critical eye surgery was denied.) The problem of limited access is compounded by the fact that patients infected with HIV are often members of marginalized or criminalized groups (intravenous drug users, commercial sex workers, and men who have sex with men).

## Systemic treatment with valganciclovir

Systemic treatment regimens for CMV retinitis are complex and burdensome with the single exception of valganciclovir, a well-absorbed oral pro-drug of ganciclovir, the "gold standard" for treatment. Valganciclovir gives blood levels equivalent to intravenous ganciclovir, is equally effective [22], and has been the foundation for treatment of CMV retinitis in Western countries for a decade.

Treatment with intravenous ganciclovir is expensive because it requires hospitalization. With the financial incentives that are inherent in the current Chinese health care system, patients may be encouraged down the path of hospitalization until their money runs out, at which time treatment may end prematurely and be inadequate.

Outpatient treatment with valganciclovir is far more feasible, but cost is again the issue. In China at this time, the cost of a typical course of treatment for AIDS-related CMV retinitis with valganciclovir is approximately equivalent to the cost of purchasing 2 new small cars.^1 ^Valganciclovir is chemically similar to acyclovir, an inexpensive and widely available medication, so there is little inherent justification for exorbitant pricing based on the cost of drug production. This is a sad and unwelcome echo from a decade ago, when pricing of antiretroviral treatments kept medication costs beyond the reach of patients dying of treatable HIV/AIDS [23]. In Thailand, the same issue was resolved with another opportunistic infection, cryptococcal meningitis, where treatment with fluconazole was out of reach for most patients until generic production reduced the price by 98%, from $13. USD a day to 30 cents a day, and made fluconazole treatment universally available [24]. Valganciclovir is an essential drug for the treatment of CMV retinitis and it must be made affordable and widely available.

## The way forward

CMV retinitis causes blindness and identifies patients at extraordinary risk of mortality, in China and throughout SE Asia. All HIV-infected patients with CD4 < 100 cells/μL, with or without symptoms, should be systematically screened by retinal examination through a fully dilated pupil with an indirect ophthalmoscope. This should occur when the patient first presents for HIV care. Although it remains conceptually attractive to imagine that screening arrangements might be organized with trained ophthalmologists, in practice, after more than a decade of development of programs for management of patients with HIV/AIDS in China and SE Asia, there is still rarely access to ophthalmologists for CMV screening in a timely fashion. With proper training and support, retinal screening for CMV retinitis can be performed by the AIDS clinicians, and treatment can be provided with oral valganciclovir, with ophthalmologists providing care for the complications of CMV retinitis, such as retinal detachment, and IRU. Systemic treatment with valganciclovir for patients diagnosed with early disease affords the best opportunity to reduce blindness and mortality from this neglected disease.

## Endnotes

^1^One bottle of valganciclovir, containing 60 × 450 mg tablets, currently costs ~17,000 Renminbi (RMB) or ~2625 USD. A typical anti-CMV treatment regimen would be 900 mg twice a day for three weeks, followed by 450 mg twice a day for 9 weeks. This would require 210 pills (3.5 bottles) at a cost of 59,500 RMB. A basic 2011 model of a popular small car in China called 'Chery QQ3' can be purchased for <30,000 RMB.

## Competing interests

The authors declare that they have no competing interests.

## Pre-publication history

The pre-publication history for this paper can be accessed here:

http://www.biomedcentral.com/1471-2334/11/327/prepub
